# Evaluation of different magnetic resonance imaging contrast materials to be used as dummy markers in image-guided brachytherapy for gynecologic malignancies*

**DOI:** 10.1590/0100-3984.2015.0004

**Published:** 2016

**Authors:** Camila Pessoa Sales, Heloisa de Andrade Carvalho, Khallil Chaim Taverna, Bruno Fraccini Pastorello, Rodrigo Augusto Rubo, Arthur Felipe Borgonovi, Silvia Radwanski Stuart, Laura Natal Rodrigues

**Affiliations:** 1 PhD, Medical Physicist, Radiotherapy Department, Instituto de Radiologia do Hospital das Clínicas da Faculdade de Medicina da Universidade de São Paulo (InRad/HC-FMUSP), São Paulo, SP, Brazil.; 2 PhD, Medical Coordinator of the Radiotherapy Department, Instituto de Radiologia do Hospital das Clínicas da Faculdade de Medicina da Universidade de São Paulo (InRad/HC-FMUSP), São Paulo, SP, Brazil.; 3 MSc, Medical Physicist, LIM/44 - Laboratório de Ressonância Magnética em Neurorradiologia, Department of Radiology and Oncology, Faculdade de Medicina da Universidade de São Paulo (FMUSP), São Paulo, SP, Brazil.; 4 PhD, Medical Physicist, LIM/44 - Laboratório de Ressonância Magnética em Neurorradiologia, Department of Radiology and Oncology, Faculdade de Medicina da Universidade de São Paulo (FMUSP), São Paulo, SP, Brazil.; 5 MSc, Medical Physicist, Radiotherapy Department, Instituto de Radiologia do Hospital das Clínicas da Faculdade de Medicina da Universidade de São Paulo (InRad/HC-FMUSP), São Paulo, SP, Brazil.; 6 BA, Medical Physicist at Royal Philips Electronics, Eindhoven, the Netherlands.; 7 MD, Physician in the Division of Radiotherapy, Department of Radiology and Oncology, Faculdade de Medicina da Universidade de São Paulo (FMUSP), São Paulo, SP, Brazil.

**Keywords:** Imaging, three-dimensional, Brachytherapy/methods, Diagnostic techniques, obstetrical and gynecological, Magnetic resonance imaging/methods, Contrast media

## Abstract

**Objective:**

To identify a contrast material that could be used as a dummy marker for magnetic resonance imaging.

**Materials and Methods:**

Magnetic resonance images were acquired with six different catheter-filling materials-water, glucose 50%, saline, olive oil, glycerin, and copper sulfate (CuSO_4_) water solution (2.08 g/L)-inserted into compatible computed tomography/magnetic resonance imaging ring applicators placed in a phantom made of gelatin and CuSO_4_. The best contrast media were tested in four patients with the applicators in place.

**Results:**

In T2-weighted sequences, the best contrast was achieved with the CuSO_4_-filled catheters, followed by saline- and glycerin-filled catheters, which presented poor visualization. In addition (also in T2-weighted sequences), CuSO_4_ presented better contrast when tested in the phantom than when tested in the patients, in which it provided some contrast but with poor identification of the first dwell position, mainly in the ring.

**Conclusion:**

We found CuSO_4_ to be the best solution for visualization of the applicator channels, mainly in T2-weighted images *in vitro*, although the materials tested presented low signal intensity in the images obtained *in vivo*, as well as poor precision in determining the first dwell position.

## Abstract (pt)

**Objetivo:**

Encontrar um material de contraste que possa ser utilizado como fonte falsa em imagens de ressonância magnética.

**Materiais e Métodos:**

Foram feitas imagens de ressonância magnética de cateteres preenchidos com seis meios de contraste conhecidos: água, glicose 50%, soro fisiológico, óleo de oliva, glicerina, solução aquosa de sulfato de cobre (CuSO_4_) (2.08 g/L), inseridos em aplicadores de sonda e anel compatíveis com tomografia computadorizada/ressonância magnética colocados em um objeto simulador de gelatina e CuSO_4_. Os materiais com melhores respostas foram testados em quatro pacientes que utilizaram os aplicadores.

**Resultados:**

Os cateteres preenchidos por CuSO_4_ apresentaram melhor visualização em sequências T2, seguidos pelos cateteres com solução de soro fisiológico e glicerina, que apresentaram visualização insatisfatória. O CuSO_4_ apresentou bom contraste em sequências T2 quando testado em objeto simulador e em pacientes, porém com uma identificação insatisfatória da primeira posição de parada possível para fonte, principalmente no anel.

**Conclusão:**

O CuSO_4_ se mostrou a melhor solução para visualização dos canais dos aplicadores, principalmente em sequências T2 *in vitro*, mas os materiais testados apresentaram baixo sinal nas imagens de ressonância magnética *in vivo* e uma precisão insatisfatória para identificação da primeira parada de fonte possível.

## INTRODUCTION

The major challenge in radiation therapy is to treat lesions with a high effective dose, while minimizing the dose to adjacent normal tissue and organs at risk^(1)^, thus diminishing side effects and treatment complications. To achieve that goal, new technologies have been developed for treatment planning and dose delivery, including three-dimensional (3D) radiotherapy, intensity-modulated radiotherapy, tomotherapy and rapid arc techniques^(2-4)^. Because of its high dose-gradient characteristic, brachytherapy has the potential to help radiation oncologists achieve a good therapeutic ratio (a high dose to the tumor with good preservation of the surrounding normal tissue). In cancer of the uterine cervix, which is one of the most common forms of cancer among women worldwide, brachytherapy plays a major role in local control and patient survival^(5)^. Image-guided or 3D brachytherapy for gynecologic malignancies has the potential to improve local control and survival even further among cervical cancer patients^(6,7)^. However, such techniques are still not in routine use, even in developed countries^(8,9)^.

For 3D brachytherapy planning, it is necessary to conduct image studies that allow volumetric reconstruction of the tumor (or target) and organs at risk. In general, computed tomography (CT) is the method of choice. However, for gynecologic tumors, magnetic resonance imaging (MRI) is the best method to assess the primary tumor volume. Various groups have been studying the use of CT or MRI for 3D brachytherapy planning^(6-10)^, in order to treat the appropriate target volume and quantify the dose delivered to the organs at risk (rectum and bladder), with promising results.

High-dose-rate brachytherapy 3D planning requires applicator reconstruction, which is primarily based on the first dwell position of the source, and should be as precise as possible. For this purpose, dummy seeds are usually placed inside the applicators during image acquisition. The metallic dummies supplied by the manufacturers are CT-compatible but are not MRI-compatible. Since tumor visualization on CT images is limited, implementation of a complete 3D treatment strategy involving delineation of the tumor volume (MRI-based) and of the volume of the organs at risk (MRI- or CT-based) is still a challenge in current practice.

The purpose of this study was to evaluate materials that may be used as contrast media for MRI and verify if they can be used as dummy markers for gynecologic 3D brachytherapy planning.

## MATERIALS AND METHODS

**Figure 1:**
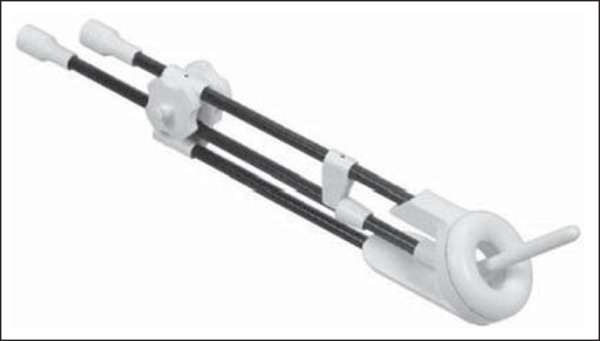
CT-/MRI-compatible ring applicator for gynecologic malignancies.

Six capillary catheters were manually filled with six different solutions to be tested: water, glucose 50%, saline, glycerin, olive oil, and a copper sulfate (CuSO_4_) water solution (2.08 g/L)^(11)^, and their extremities were sealed with wax. A phantom composed of gelatin and CuSO_4_^(11)^ was constructed specifically for image acquisition. As depicted in Figure 1, CT-/MRI-compatible ring applicators (Nucletron/Elekta AB, Stockholm, Sweden) were placed in the gelatin and the catheters were inserted in the applicators.

To determine which solution and image acquisition technique would provide better visualization of the applicator lumen, T2-weighted images were acquired with variation of the acquisition parameters in a 3 T scanner (Achieva; Philips Healthcare, Eindhoven, the Netherlands) and in a 1.5 T scanner (Signa HDxt; GE Healthcare, Chalfont St. Giles, UK) scanners. The 1.5 T scanner is the default equipment for patient imaging. The acquisition parameters were sequence type, echo time (TE), repetition time (TR), slice spacing, and slice thickness (Table 1). The slices were aligned following the tandem axis, parallel to the ring, according to the applicator geometry. Despite the possibility of obtaining good contrast on T1-weighted sequences with gadolinium, the tests were conducted only on T2-weighted sequences, because this is the standard for delineation of gynecologic tumors^(12)^.

**Table 1:** Parameters used in MRI sequences.

			Slice			
			Spacing	Thickness	TR	TE
Series	Orientation	Sequence	(mm)	(mm)	(ms)	(ms)
1	Coronal	T2 Cube	1.3	1.3	1500	140
2	Axial	T2 Cube	1.3	1.3	1500	140
3	Oblique	T2 Cube	1.3	1.3	1500	140
4	Axial	T2 FSE	0.5	3.0	5083	140
5	Axial	T2 FSE	1.0	3.0	7000	140
6	Axial	T2 FSE	1.5	1.5	9000	140

FSE, fast spin-echo.

A brachytherapy treatment planning system (Oncentra Master Plan; Elekta AB, Stockholm, Sweden) was used in order to analyze the images visually. The best sequence to visualize the dummies was determined through qualitative analysis of the images, and the process was performed by two observers in order to minimize its variability. Subsequently, the solutions that, in a qualitative analysis, presented the best contrast in the phantom were tested in four patients in whom 1.5 T MRI was performed with the brachytherapy applicators in place. The study was approved by the local institutional review board and research ethics committee.

## RESULTS

**Figure 2:**
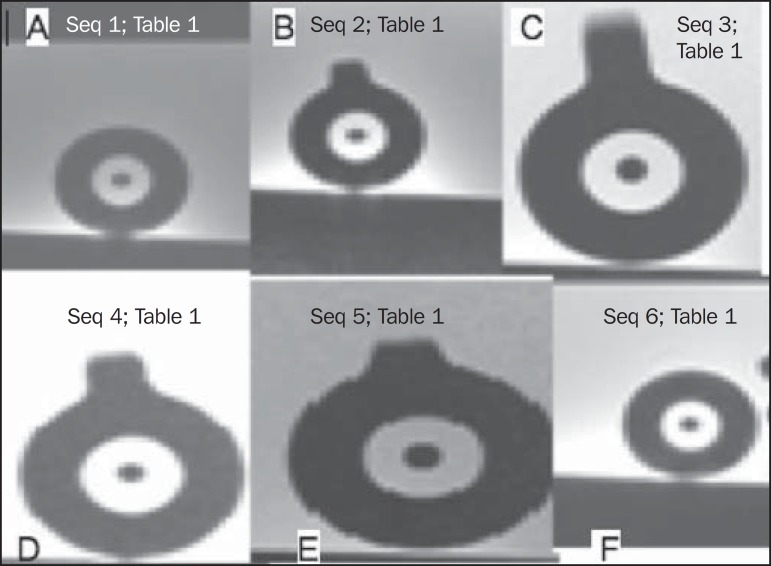
MRI scan (T2-weighted sequence) in which the glycerin ring lumen and saline tandem lumen solutions were tested as dummies. The figures show the various acquisitions (detailed in Table 1) for each solution.

**Figure 3:**
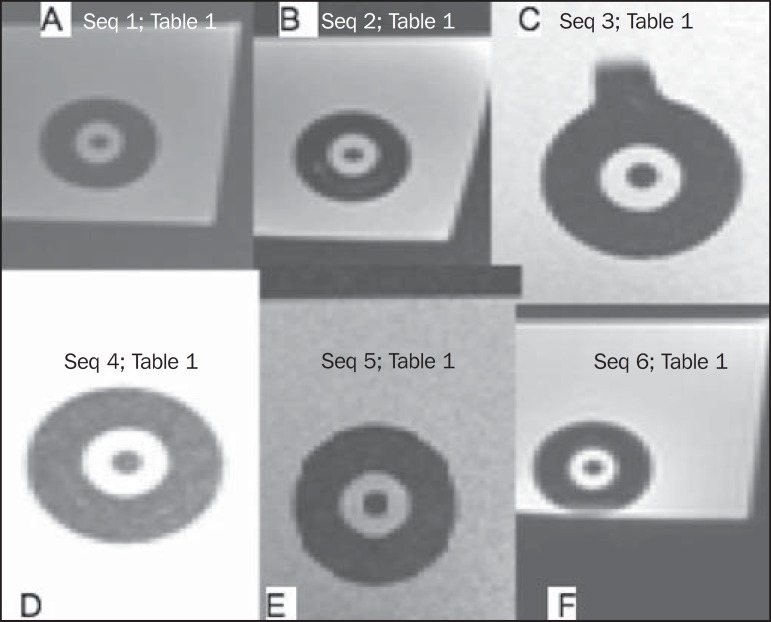
MRI scan (T2-weighted sequence) in which the CuSO_4_, ring lumen and tandem lumen solutions were tested as dummies. The figures show the various acquisitions (detailed in Table 1) for each solution.

**Figure 4:**
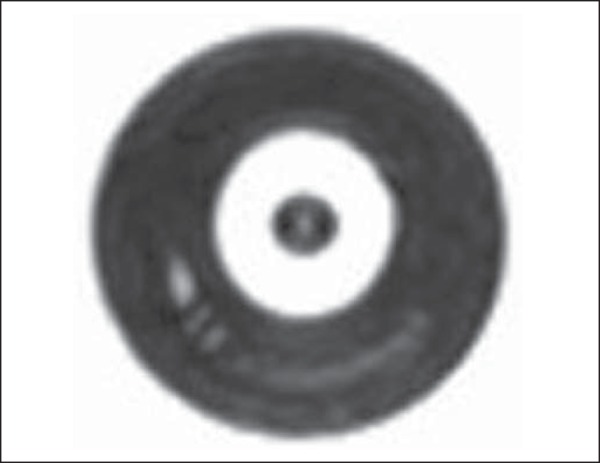
MRI scan (T2-weighted sequence) in which the CuSO_4_ dummy is visualized in the ring lumen.

**Figure 5:**
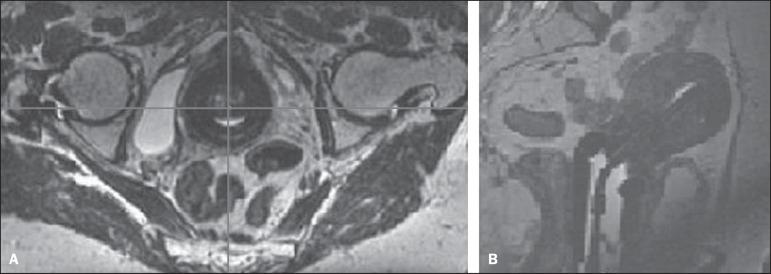
MRI scan (T2-weighted sequence) of one patient with the applicators in place and the CuSO_4_ dummies. **A:** Axial section: ring. **B:** Sagittal section: tandem.

The images were compared in order to determine which solution and MRI sequence promoted the best visualization for applicator reconstruction (Figures 2 and 3). In T2-weighted sequences, the CuSO_4_ solution presented the highest contrast (Figure 3), followed by glycerin and saline, both of which presented poor contrast (Figure 2). Comparing these solutions, CuSO_4_ presented a better contrast than saline and glycerin. The best combination was an axial T2-weighted fast spin-echo cube sequence-slice spacing = 1.3 mm, slice thickness = 1.3 mm, TR = 1500 ms, and TE = 140 ms (Table 1)- used in order to visualize the CuSO_4_ solution (Figure 4). Efforts to place a specific slice as the most coincident with the dummy in the ring had to be made in order to achieve better visualization of the dummy. When tested in patients, CuSO_4_ presented some contrast in T2-weighted sequences (Figure 5). Although CuSO_4_ could be visualized in the MRI sequences obtained in the patients (Figure 5), the resolution was not sufficient to determine the first dwell position, mainly in the ring.

## DISCUSSION

Image-guided brachytherapy for gynecologic malignancies can be performed with CT or MRI scans. However, T2-weighted MRI is the gold standard for defining the tumor volume in patients with cancer of the uterine cervix^(12)^. When the procedure is performed exclusively on the basis of CT scans, tumor coverage is not evaluated appropriately. Nevertheless, applicator reconstruction is a key point for a precise and definitive calculation, and manufacturers do not provide MRI-compatible dummies for all kinds of applicators. One option to solve this problem is CT-MRI image fusion, although that leads to uncertainties due to image distortions that may be unacceptable, mainly in brachytherapy. In addition, the need to perform two image studies for the same procedure raises the costs, which cannot be neglected. Therefore, applicator reconstruction in MRI scans, where the target volume is best visualized, is mandatory for complete 3D treatment.

The CuSO_4_ solution provided a good signal, with an acceptable, albeit poor, applicator reconstruction, mainly due to the difficulty in identifying the ends of the catheters. The wax material used to seal the catheter tips does not provide good MRI contrast. In addition, because the catheters are very thin, high precision is needed in order to seal the tips with an exact, known amount of wax, which was not achieved in this experiment. Haack et al.^(11)^ reported that CuSO_4_ provides a degree of MRI contrast better than that demonstrated in our study. Because those authors used applicators from a different manufacturer, it is likely that the difference between the two types of applicators, in terms of the caliber of the channels (Nucletron applicators have a narrower lumen) could explain the lower contrast that we observed. All of the other materials tested in our study also presented low signal intensity in the MRI scans. This was, once again, probably due to the very narrow applicator lumen, allowing only a capillary-like catheter to be inserted, with a very low volume of contrast. Recently, the manufacturer developed a new applicator with a larger lumen, which could improve the visualization of contrast inside larger catheters, although only a few hospitals in developing countries can afford to purchase this new applicator.

Only three of the six solutions tested were visible in the T2-weighted images generated using the phantom, and only one allowed satisfactory visualization. However, when used in the patients, the contrast was insufficient to reconstruct the applicators appropriately. The differences found between the images generated using the phantom and those acquired in the patients were probably due to the variety of signals generated by the patients themselves, which can make it difficult to identify low-intensity signals like those emanating from very thin catheters.

As an alternative to solve the problem of applicator reconstruction, a radiograph of the rings with the dummies provided by the manufacturer (that are only CT-compatible) may be printed in a transparency^(13)^. This is superimposed to the ring in an MRI scan in the same projection as the X-ray. For the tandem, the procedure is much easier, since the distance from the first dwell position to the tandem tip is already known, which allows a very reliable reconstruction to be performed.

The best alternative for applicator reconstruction is the "applicator library" provided by the manufacturer for tandem and ring applicators^(14)^. With this tool, it is possible to estimate the location of the first dwell position. The precise determination of this location is not mandatory when using the library, because it is possible to match the entire applicator library with the MRI applicator.

**Figure 6:**
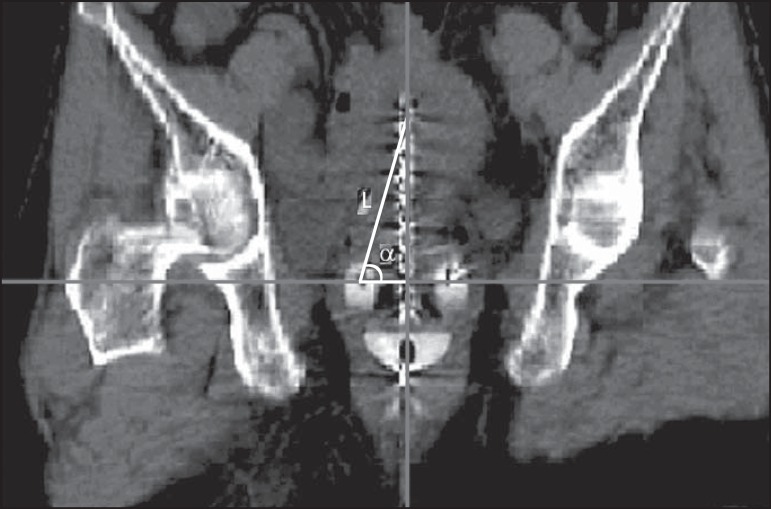
Coronal section showing the distance between the first dwell position in the tandem and the ring (L), as well as the angle (α) between this distance and the applicator origin.

**Figure 7:**
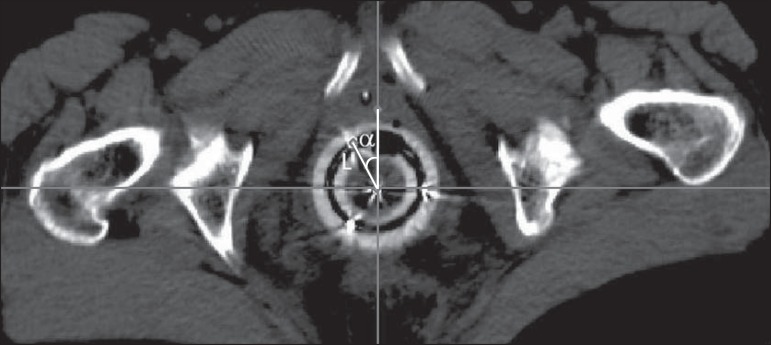
Axial section image showing the distance (L) between the first dwell position in the ring and the applicator origin, as well as the angle (α) between this distance and the axis perpendicular to the rectal retractor, when the image is aligned with the rectal retractor in this plane, to verify the ring rotation.

A valid option for evaluating the precision of the reconstruction is to determine the distance from the tip of the tandem and the ring to the first dwell position, as an initial reference point for reconstruction in the images. This also can be a challenge, mainly in relation to the ring, because this reference should be based in the acquired images. In order to evaluate the reliability of the applicator reconstruction, it is recommended that the fixed applicator distances and angles determined previously be compared with those measured in MRI^(11)^. The parameters of interest are the distance between the first dwell position in the tandem and the ring, together with the angle between this distance and the applicator origin, in the coronal section (Figure 6), as well as the distance between the first dwell position in the ring and the applicator origin, together with the angle between this distance and the axis perpendicular to the rectal retractor, in the axial section when the image is aligned with the rectal retractor to verify the ring rotation (Figure 7).

Comparing the different contrast solutions, we were able to identify the best contrast media to be used in MRI. However, with the applicator studied, the results were not satisfactory. The contrast solution may be an option to be used in other situations in which determination of the first dwell position is mandatory, as when different types of applicators, such as those with a larger lumen, are used.

## CONCLUSIONS

We found CuSO_4_ to be the best solution for *in vitro* visualization of the applicator channels, mainly in T2-weighted images. However, the materials tested presented low signal intensity in the *in vivo* images and poor precision in determining the first dwell position, thus precluding appropriate reconstruction, since the applicators studied have a very narrow channel diameter and the dummy manufacturing process used was not suitable. For clinical purposes, it is necessary to use the library of applicators to guarantee an accurate reconstruction.
